# Refractory kaposiform lymphangiomatosis relieved by splenectomy

**DOI:** 10.3389/fped.2023.1203336

**Published:** 2023-08-17

**Authors:** Yuru Lan, Jiangyuan Zhou, Tong Qiu, Xue Gong, Yi Ji

**Affiliations:** Department of Pediatric Surgery, West China Hospital of Sichuan University, Chengdu, China

**Keywords:** kaposiform lymphangiomatosis, splenectomy, angiopoietin 2, platelet, angiopoietin 1

## Abstract

**Introduction:**

Kaposiform lymphangiomatosis (KLA) is a rare and complex lymphatic anomaly with a poor prognosis. There is no standard treatment, and drug therapies are the most common therapeutic method. However, some patients' symptoms become gradually aggravated despite medical treatment. Splenectomy may be an alternative option when pharmacological therapies are ineffective.

**Materials and Methods:**

We reviewed and evaluated the cases of 3 patients with KLA who ultimately underwent splenectomy. Results: The lesions were diffusely distributed and involved the lungs and spleens of the 3 patients. Laboratory examinations revealed that all three patients had thrombocytopenia and reduced fibrinogen levels. All patients underwent symptomatic splenectomy after the medication failed. Surprisingly, their symptoms greatly improved. Histopathological investigation of the splenic lesions of the three patients confirmed the diagnosis of KLA. Immunohistochemical staining showed positivity for CD31, CD34, podoplanin, Prox-1 and angiopoietin 2 (Ang-2).

**Discussion:**

This study aimed to review the features of KLA patients treated by splenectomy and explore the underlying link between splenectomy and prognosis. The reason for the improvement after splenectomy may be related to increased Ang-2 levels and platelet activation in patients with KLA. Future research should seek to develop more targeted drugs based on molecular findings, which may give new hope for the treatment of KLA.

## 1. Introduction

Kaposiform lymphangiomatosis (KLA), which is a novel subtype of a generalized lymphatic anomaly (GLA), is a rare and devastating lymphatic anomaly (1–3). It often occurs in children, and the first reported case series found that the mean interval time from diagnosis to death was 2.75 years and the 5-year survival was 51% (1). KLA exhibits a complex, diverse, and long-term course. The mediastinum, lung, and pleura are the prevalent sites of intrathoracic involvement, while the spleen and bones are the frequently affected extrathoracic sites (1, 4, 5). Owing to its various and vague symptoms, the diagnosis and confirmation are difficult and can easily be delayed, leading to poor prognosis and significant mortality.

There is no standard treatment, and the appropriate treatment regimen remains controversial. Previously reported pharmacotherapies, which are used alone or in combination, include vincristine, sirolimus, interferon-alpha, corticosteroids, and trametinib (5). However, patients with KLA have variable responses to pharmacotherapies. Almost half of KLA cases may involve the spleen (1). Splenectomy may be considered in severely affected patients with life-threatening KLA and splenic infiltration and has been reported to ameliorate coagulation and hematological parameters (4, 6–8).

Here, we report three KLA patients with pulmonary symptoms; the patients underwent splenectomy after ineffective medical treatment. We reviewed the clinical and pathological features of these three cases, with the goals of better understanding the natural history of KLA and exploring the mechanism of the positive effect of splenectomy on prognosis.

## 2. Materials and methods

This study was performed after obtaining permission from the Institutional Review Board of West China Hospital of Sichuan University. The parents of all patients gave written informed consent.

The study reviewed 20 patients diagnosed with KLA between April 2018 and December 2022. The diagnosis was based on clinical data, imaging studies, and biopsy (1, 6, 9). The following are the inclusion criteria: the diagnosis was confirmed by pathological examination, and a splenectomy was performed. The patients were excluded if detailed clinical data were absent or insufficient. A total of four patients underwent splenectomy. Due to the loss of follow-up, one patient was not included in this study; so finally, we included three patients. For pathological comparison, the spleens of the patients with traumatic splenic rupture were included. The children with traumatic splenic rupture received a partial splenectomy. We analyzed the spleen tissues of the KLA patients and the spleen tissues of the children with traumatic splenic rupture. Tissue sections were stained with hematoxylin and eosin. Immunohistochemistry followed our previous process (10). Primary antibodies against CD31 (1:800, CST#3528, Cell Signaling Technology), CD34 (1:50, CST#3569, Cell Signaling Technology), podoplanin (1:400, 11629-1-AP, Proteintech), Ki67 (1:800, CST#9449, Cell Signaling Technology), *α*-SMA (1:400, CST #19245, Cell Signaling Technology), and angiopoietin 2 (Ang-2) (1:50, ET1705-6, HUABIO) were added. These tissue sections were incubated overnight at 4°C. The slides were washed and incubated with secondary antibodies for 30 min at room temperature. The images were obtained through a microscope camera (Leica Microsystems, Wetzlar, Germany).

## 3. Results

### 3.1. Baseline characteristics

A total of three KLA patients, consisting of two males and one female who all underwent splenectomy, were included in the study. The age of onset ranged from 9.0 to 43.0 months, with an average of 21.3 months. All patients had respiratory symptoms of varying degrees, including cough, shortness of breath, dyspnea, and even pneumonia. Two patients had pleural effusion, and two patients had pericardial effusion. One patient had gastrointestinal bleeding at the time of presentation. The characteristics of the patients, consisting of basic information, treatment methods, and prognosis, are summarized in Table 1.

**Table 1:** A summary of the general information, treatment intervention, and prognosis of patients with KLA.

Cases	Sex	Age at presentation (months)	Age at diagnosis (months)	Location	Types of lesions	Major symptoms, signs, and/or complications	Previous pharmacotherapy (months)	Postoperative outcome
1	Female	12	18	Neck, mediastinum, lung, spleen	Diffuse	Tachypnea, pericardial effusion, pneumonia, splenomegaly	Sirolimus plus prednisolone (3.0)	Improved
2	Male	9	13	Lung, spleen	Diffuse	Cough, respiratory distress, pericardial effusion, pleural effusion, pneumonia, splenomegaly	Sirolimus (4.5), trametinib (3.0), and prednisolone (1.5)	Improved
3	Male	43	52	Neck, mediastinum, lung, spleen	Diffuse	Cough, respiratory failure, gastrointestinal bleeding, severe pneumonia, pericardial effusion, pleural effusion, splenomegaly, disseminated intravascular coagulation	Sirolimus (1.5) and Ureidopenicillin (1.5)	Improved

All patients were first treated regularly with medication. All patients achieved therapeutic levels of sirolimus during the attempted treatment period. The starting dose of oral sirolimus was 0.8 mg/m^2^ administered twice daily. Subsequently, oral sirolimus was titrated to achieve trough levels of 10–15 ng/ml. Prednisolone was administered at 2 mg/kg orally once daily. Trametinib was administered at 0.1 mg/kg orally once daily. However, the medication treatment was ineffective. Persistent breathing difficulties in the three patients were recorded. All patients had sustained clinical deteriorations in their platelet counts, even after receiving pharmacological treatment. The conditions of the patients were severe, and splenectomy was recommended to save their lives. All patients received a 13-valent pneumococcal conjugate vaccine, the quadrivalent (ACWY) meningococcal and *Haemophilus influenzae* type b vaccination, before surgery. Medications were discontinued after splenectomy, and all patients underwent regular follow-up after surgery.

Here, we report Patient 1 in detail. The child was a 12-month-old female who was admitted due to tachypnea. Then, she was diagnosed with KLA after admission and evaluation of her medical history and imaging examination. Because the child had dyspnea and a low platelet count, she was given sirolimus (0.8 mg/m^2^, po, bid) combined with prednisolone (2 mg/kg, po, qd). During the medication treatment process, the platelet count of the patient briefly increased but quickly decreased. In addition, she had orthopnea accompanied by a large amount of pericardial effusion. To alleviate the symptoms, pericardiocentesis was performed to drain a total of 355 ml of bloody fluid. There was a slight improvement in her respiratory symptoms after the surgery. However, many lesions in the chest cavity did not improve during the medication period. The platelet count of the patient continued to decrease, and her breathing was difficult. Due to the progression of her condition and ineffective drug treatment, the patient ultimately underwent splenectomy. The postoperative recovery of the child was remarkable, with an increased platelet count and stable vital signs. Two weeks after the surgery, the platelet count of the patient reached its peak at 1,035 × 10^9^/L, and the D-dimer level also returned to normal at 2.57 mg/L. Afterward, the follow-up examinations were conducted at regular intervals. The platelet counts of the patient were slightly higher than normal, and the D-dimer levels were normal. The MRI images at each follow-up also indicated that the postoperative chest lesions in the patient were alleviated, but there were occasional inflammatory manifestations.

The condition of the other two children was similar, but the type and duration of medication varied depending on the situation. Patient 2 was given sirolimus, prednisolone, and trametinib. However, the patient failed to achieve the expected effect, and the disease progressed rapidly. Patient 3 had severe respiratory symptoms and abdominal distention. He received sirolimus monotherapy and several symptomatic treatments to ease his symptoms (e.g., multiple gastrointestinal decompressions). The treatment remained difficult because these two patients showed no responses to medical therapies. Patient 2 had dyspnea and a large amount of pleural effusion during the disease course. Patient 3 was unable to maintain oxygen saturation and had significant dyspnea during the medication process. After a tracheal intubation, an invasive ventilation was performed. At the same time, the coagulation function of the patient could not be corrected, and it was speculated to be related to splenic lesions. Pleural effusion in Patient 2 and Patient 3 required drainage to relieve the symptoms. The thoracic liquid discharged was milky white or hemorrhagic. Patient 3 also had drainage of pericardial effusion. To alleviate these symptoms, there was an urgent need to apply an alternative plan. There were numerous nodular lesions in the spleen with splenomegaly. Combined with previous medication and splenic lesions, a multidisciplinary discussion regarding the disease and treatment plans of the three patients led the team to recommend splenectomy. The postoperative recovery of all patients was good, and follow-up is ongoing.

### 3.2. Imaging features

**Figure 1:**
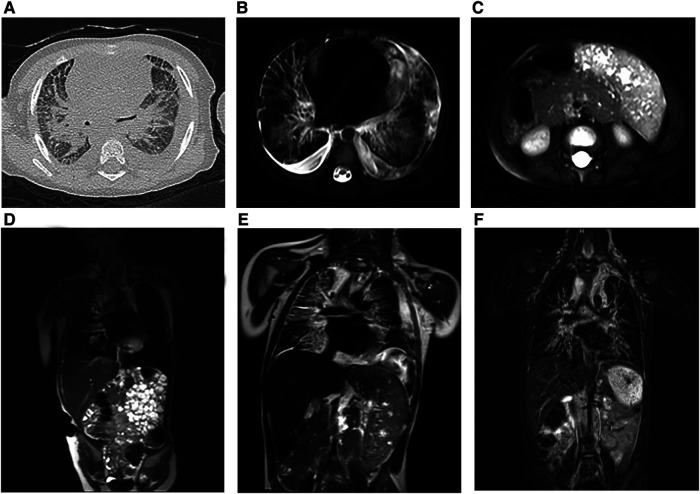
Preoperative radiologic findings in KLA (Patients 1–3). (**A**) (Case 2) The CT scan image showed multiple patches and stripes and suspicious thickening of interlobular septa in both lungs with bilateral pleural effusion. (**B**) (Case 2) Horizontal T2-weighted MRI of the chest revealed scattered striped abnormalities and pericardial effusion. (**C**) (Case 2) Horizontal T2-weighted MRI showed enlargement of the spleen and multiple nodular lesions in the spleen parenchyma. (**D**) (Case 1) Coronal T2-weighted MRI images showed bilateral hilar enlargement, no enlargement of the hilar and mediastinal lymph nodes, and no displacement of the mediastinum. The heart was enlarged with a small amount of pericardial effusion. (**E**) (Case 2) Coronal T2-weighted MRI showed that the soft tissue shadows in the cervical root, mediastinum, and right hilar area increased; the boundary was unclear; the bronchial wall in the upper and lower lobes of the right lung was thickened; and the interlobular septa of the right lung was thickened. (**F**) (Case 3) Significant interstitial changes in both lungs were accompanied by a large amount of pleural effusion on the left side and a small amount of pleural effusion on the right side. The spleen was enlarged, the lower margin reached the pelvic cavity, and numerous nodules could be seen in it (coronal T2-weighted MRI).

All patients underwent detailed imaging examinations, both for preoperative and postoperative evaluation. Computed tomography (CT) and magnetic resonance imaging (MRI) are the main methods of auxiliary modes. KLA was characterized by infiltrating soft tissue thickening, low-attenuation mass, or different degrees of pleural effusion on CT (Figure 1A), and MRI showed interstitial thickening and mediastinal infiltration (Figure 1B). When the lesion involved the spleen, the volume of the spleen usually increased, and there were many abnormal nodule signal foci (Figure 1C). The lesions of the three patients were diffuse with varying degrees of involvement of the lung and spleen (Figures 1D–F).

### 3.3. Laboratory examinations

In this study, we regularly monitored the routine hematological parameters and coagulation function of all patients. Three patients had fibrinogen reduction, and the lowest concentration was 0.56 g/L. All patients had thrombocytopenia, and the lowest platelet count was 13 × 10^9^/L. The detailed monitoring data can be found in Table 2.

**Table 2:** Summary of hematological parameters of patients with KLA at the time of the lowest recorded platelet count and at 2 weeks after splenectomy.

Cases	PLT (×10^9^/L)		WBC (×10^9^/L)		HGB (g/L)		FIB (g/L)		D-dimer (mg/L)		PT (s)		APTT (s)	
	Pre	After	Pre	After	Pre	After	Pre	After	Pre	After	Pre	After	Pre	After
1	74	1,035	24.91	10.95	109	140	0.56	3.21	11.60	2.57	13.1	10.6	26.4	25.4
2	55	1,156	11.58	11.51	125	108	1.12	2.47	20.39	–	14.7	12.3	43.8	36.4
3	13	1,009	12.4	9.5	58	115	1.41	2.30	–	–	150	10.7	300	26.6

PLT, platelet; WBC, white blood cell; HGB: hemoglobin; FIB, fibrinogen; PT, prothrombin time; APTT, activated partial thromboplastin time.

### 3.4. Pathological findings

**Figure 2:**
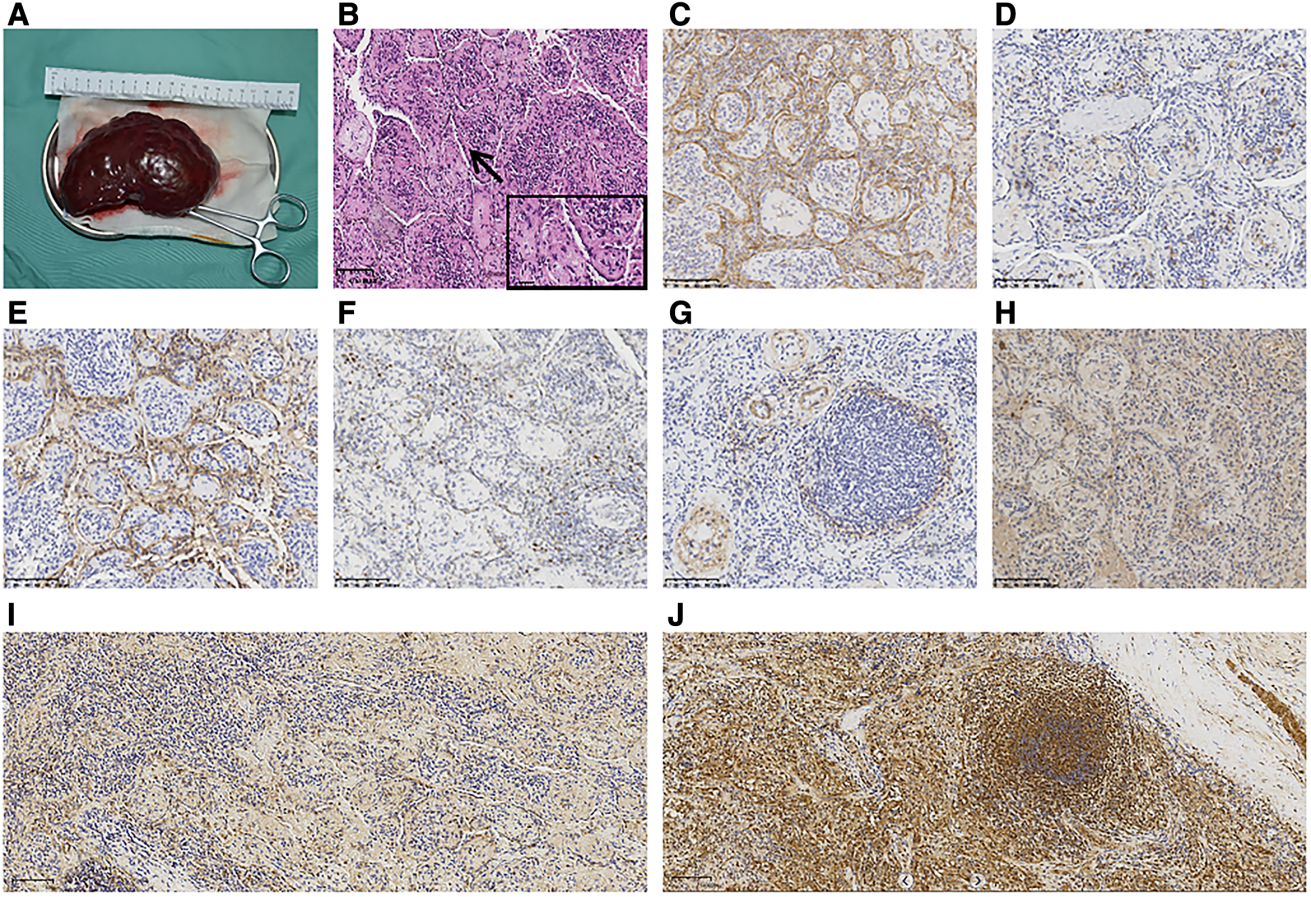
Pathological results of KLA with lung and spleen involvement (Patient 1). (**A**) Macroscopic view of the spleen excised from the patient. (**B**) Diffuse proliferation of abnormal, dilated lymphatics and small fascicles of hemosiderin-laden spindled lymphatic endothelial cells (HE). (**C–E**) Spindle-like cells were strongly immunopositive for CD31 (**C**), CD34 (**D**), and podoplanin (**E**). (**F**) Slight positive staining of Ki67. (**G**) *α*-SMA was focally positive and mainly concentrated in the blood vessel wall around the tumor focus (**G**). (**H**) Prox-1 was strongly positive. (**I**) Negative staining of spleen tissue in children with splenic rupture for Ang-2. (**J**) Positive staining of the spleen of KLA for Ang-2.

The spleens in the KLA patients were enlarged with macroscopic cystic spaces. We performed splenectomy on the patients. In the three cases, the splenic lesions were examined pathologically. The pathological findings are shown in Figures 2A–H. The lesions were immunopositive for CD31, CD34, podoplanin, and Prox-1. Minor immunopositivity for SMA and Ki67 was observed. Immunohistochemistry was performed on the spleen of the KLA patients, and for comparison, we also added immunohistochemical staining of Ang-2 in the spleen of the children with traumatic splenic rupture. A strong positive expression of Ang-2 was detected in the spleen of the KLA patients. Ang-2 was negative in the spleen partially removed due to traumatic rupture (Figures 2I,J).

### 3.5. Prognosis

**Figure 3:**
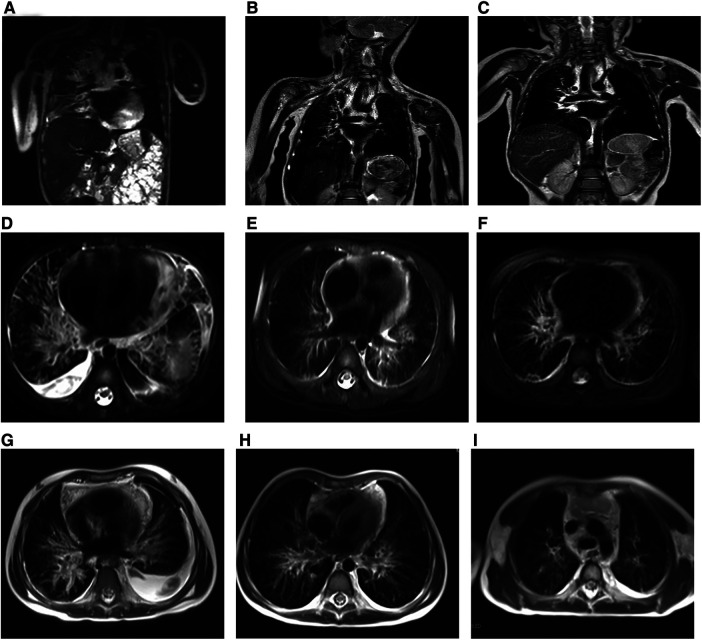
Preoperative and postoperative radiologic findings in KLA (Patients 1–3). (**A–C**) (Case 1) (**A**) Preoperative image. Coronal T2-weighted MRI of the chest revealed that 6 (**B**) and 12 months (**C**) after surgery, the inflammation of the patient improved, and the pericardial effusion decreased significantly. (**D–F**) (Case 2) Preoperative image (**D**) and postoperative images at 6 (**E**) and 12 months (**F**). The inflammation improved, and the fluid accumulation decreased compared with the previous period. All images are horizontal T2-weighted MRI images. (**G–I**) (Case 3) The presented images are all horizontal T2-weighted MRI images. The child had a large amount of pleural effusion and significant pulmonary inflammation before surgery (**G**). The symptoms of the child improved gradually, and the pleural effusion had mostly disappeared by 6 months (**H**) after the operation. The inflammation improved 12 months after surgery (**I**).

**Figure 4:**
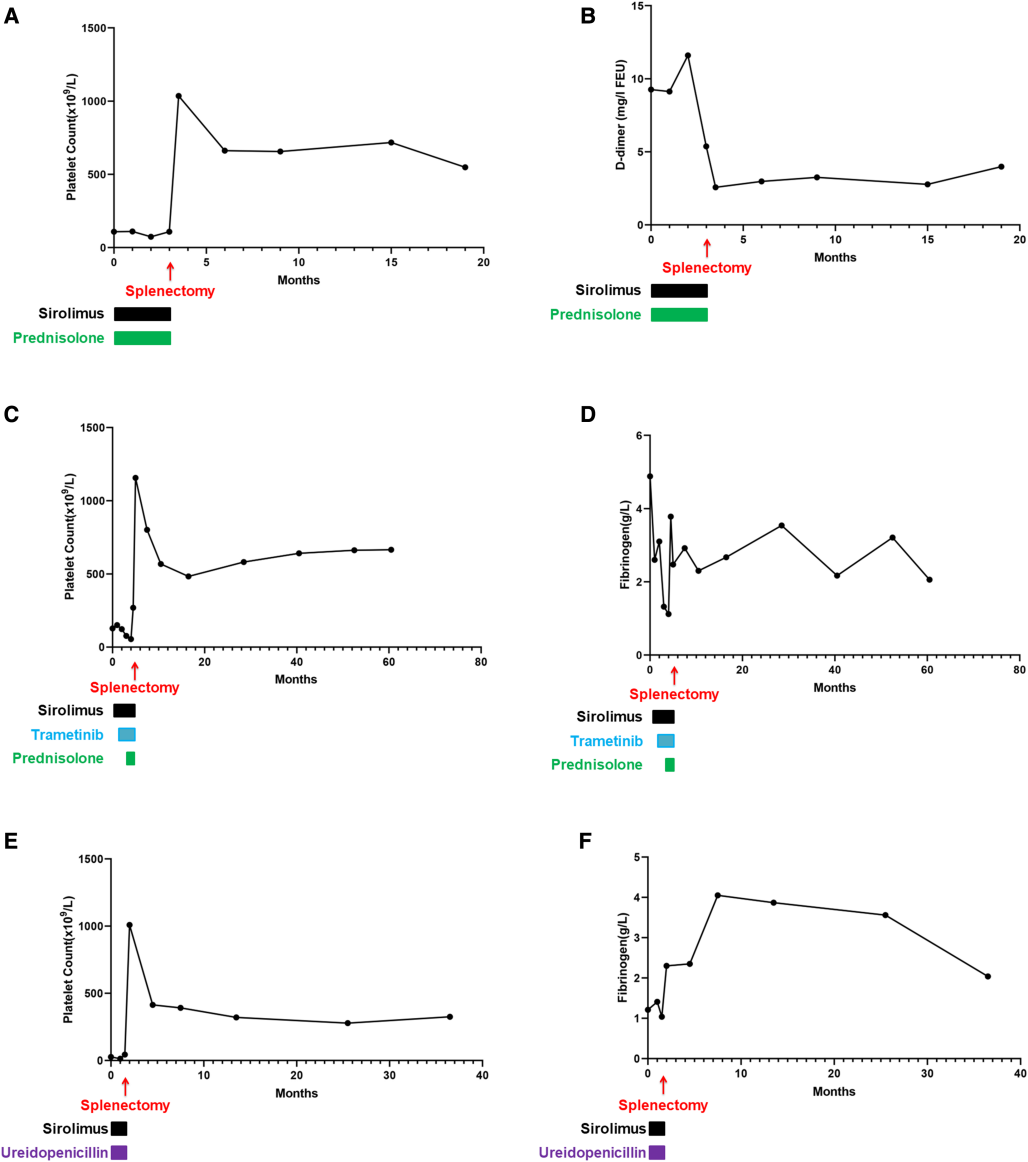
Hematology parameters of all patients throughout the disease. (**A**) Platelet count of Case 1. After surgical treatment, the index increased and fluctuated consistently in a high range thereafter. (**B**) D-dimer of Case 1. The changes in D-dimer were consistent with the trend of the platelet count changes. (**C**) Platelet count of Case 2. During medication, the platelet count did not increase. After splenectomy, the platelet count increased significantly and was maintained at a high level throughout the follow-up. (**D**) Fibrinogen of Case 2. During drug treatment, this value fluctuated outside the normal range, and after splenectomy, it fluctuated within the normal range. (**E**) Platelet count of Case 3. During the medication period, there was no increase in the platelet count, but after splenectomy, the platelet count remained elevated and remained constant. (**F**) Fibrinogen of Case 3. During postoperative follow-up, this indicator fluctuated within the normal range.

Fortunately, the conditions of the three patients improved, and they were followed up at the outpatient department weekly for 2 weeks, then every other week for 2 weeks, then monthly for 2 months, and then every 3 months thereafter or as clinically indicated. Examinations mainly included chest and abdomen MRI, routine blood tests, blood biochemical examinations, and coagulation function tests. The follow-up continued until December 2022, and the three patients were separately followed up for 56, 16, and 35 months. The chest MRI images of the three patients are shown in Figure 3. The postoperative vital signs of all patients were stable. To better track the changes in the hematology parameters of the patients before and after treatment, we collected their data throughout the entire process, from admission to discharge and to follow-up. For Patient 1, the indicators monitored were the platelet count and D-dimer level. There was no routine monitoring of D-dimer in the early stages for Patients 2 and 3, whereas fibrinogen was used for monitoring alternatively. In a subsequent follow-up, the hematological parameters of the patients remained basically normal (Figure 4).

## 4. Discussion

As an extremely rare, invasive, and diffuse lymphangitic disease, KLA exhibits malformations in the histologic appearance, and it also exhibits more invasive features, such as vascular tumors (1, 3, 11, 12). KLA usually involves the thoracic cavity, bone, retroperitoneum, and viscera, with various clinical manifestations (4, 9). The unique imaging characteristics are intrathoracic lesions with deteriorating respiratory symptoms and hemorrhagic effusions (6, 12). In our cases, all the presenting features were respiratory symptoms, including tachypnea, cough, and dyspnea.

Due to the diversity of symptoms, the diagnosis of KLA has been challenging, and there is no standardized diagnostic method. Indeed, the diagnosis of KLA is often delayed because KLA usually mimics GLA or kaposiform hemangioendothelioma (KHE) (1, 6, 13). Thus, imaging examinations are very important (4, 14–16). Imaging studies combined with medical history can help us make a clinical diagnosis. A pathological examination of the lesion is the gold standard for the diagnosis of KLA (1, 5). Under the microscope, clustered or flaky spindle lymphatic endothelial cells show abnormal expansion and proliferation, accompanied by scattered red blood cells. Hemosiderin deposits can be seen inside the cells. The immunoreactive markers podoplanin, LYVE-1, and Prox-1 are positive (17, 18).

There are no unified treatment approaches for KLA. Drug treatment is relatively common. Current research confirms that sirolimus may have a positive effect on a subset of KLA patients (12, 19–21). If patients have confirmed mutations, treatment with certain targeted drugs, such as trametinib, can be administered (12, 16, 22–24). Unfortunately, drug treatment is not always effective for KLA. The deaths of KLA patients are usually caused by cardiopulmonary failure or coagulation disorders (6, 25). Symptomatic treatments such as thoracic catheter drainage, pericardial puncture, and platelet transfusion are administered for pleural effusion, pericardial effusion, and low platelet counts. When the means of treatment are limited, splenectomy is an optional palliative treatment that also has certain effects (1, 4, 7). In recent years, we have treated a total of 20 patients. Among them, 11 patients had splenic lesions. All our patients were initially treated with medication, but four patients ultimately underwent splenectomy. Finally, we reported three patients with complete follow-up data.

The drug treatments for our three children were ineffective, and the conditions of the patients progressed rapidly. Ultimately, splenectomy was performed to save the lives of the patients. Surprisingly, during follow-up to date, we have seen an improvement in the symptoms in the three children who underwent splenectomy. Interestingly, when KLA cases were first reported, there were also three patients who underwent splenectomy. In that study, one patient experienced symptom relief, and two patients had only short-term improvement (1). Regrettably, the initial situation and prognosis of the three children who underwent splenectomy were not very detailed. The preoperative and postoperative data of our three patients supplement the prognosis of splenectomy. In addition, when considering this treatment approach, we should consider the risk of infection after splenectomy. In the previous studies, to prevent postoperative infections in these patients, vaccinations were recommended (26–30). Considering the possibility of infection in the children, it is necessary for our three children to receive vaccination before splenectomy. Because splenectomy is not the initial treatment regimen, vaccination was not originally planned. In the present study, all the three children received splenectomy after vaccination. After receiving a splenectomy, the symptoms of our patients improved remarkably and were relieved continuously. The lung inflammation of the three patients significantly reduced, the pleural or pericardial effusion decreased, the respiratory symptoms were significantly relieved, and the platelet counts increased. All three children are still being followed up. We have tried to find some links between splenectomy and continuous relief of symptoms and provide some ideas for the improvement and treatment of symptoms in KLA patients.

KLA is characterized by abnormal lymphatic vessels, mainly involving the thoracic cavity. The lymphatic system is the basis of interstitial circulation and immunity. Abnormal structure and function of the lymphatic system can lead to edema, hydrops, and infection. In the previous studies, Ang-2 was found to be elevated in the serum of KLA patients and could also be used as a serum marker to participate in the diagnosis and prognosis (31, 32). Our preliminary research in the field of vascular diseases also suggests the role of Ang-2 in the occurrence and development of diseases (33, 34). Our study revealed that Ang-2 is also strongly positive in splenic lesions. According to the previous studies and our results, we speculate that the main factors of symptom relief after splenectomy in KLA children depend, at least partially, on platelet activation and Ang-2 levels.

The platelet counts in KLA patients often decrease, and this decrease is often accompanied by fibrinogen reduction, which is similar to that observed in KHE with the Kasabach–Merritt phenomenon (KMP) (1, 5, 6, 13). The mechanisms of thrombocytopenia in patients with KLA and KHE are unknown. KMP is defined as a platelet count below 100 × 10^9^/L, accompanied by a consumptive coagulation disorder and hypofibrinogenemia (34). In the previous studies, there have been many hypotheses regarding the occurrence of KMP, mostly related to lesion sizes, platelet activation, and high expression of podoplanin (35, 36). Our previous research revealed that the larger the lesion in KHE patients, the higher the risk of developing KMP (37). The spleen lesions in all three patients were diffuse. In view of this finding, we speculated that the presence of numerous cystic lesions in the spleen of KLA patients accelerates the abnormal activation and destruction of platelets. In KLA patients with spleen involvement, multiple lesions in the spleen are accompanied by splenomegaly. In such patients, the rate of platelet destruction increases. However, the degree of thrombocytopenia in KLA cannot be explained solely by splenomegaly. A study showed a significant increase in thrombomodulin in splenic lesions of KLA, which is a sign of endothelial cell destruction (17). Under normal circumstances, platelets do not interact with the intima of vascular endothelial cells (38). However, in abnormal situations such as vascular injury, inflammation, and tumors, platelets may abnormally activate and release many factors that affect endothelial cell function. Platelets in patients with KLA adhere to the abnormal lymphatic endothelium, and platelets are abnormally activated (1, 34, 39–41). At the same time, cystic lesions in the spleen also capture platelets, and platelets that accumulate in the lesions are also abnormally activated. Many abnormally activated platelets are captured by the diseased spleen, and the serum platelet levels decrease. Abnormally activated platelets result in inflammation and leakage and even affect the abnormal production of blood vessels and lymph vessels (34, 42, 43).

Angiopoietin 1 (Ang-1) is largely stored in platelets and maintains endothelial stability during normal physiological functions (44, 45). Ang-2 antagonizes the effect of Ang-1 under normal conditions. It is stored in the Weibel–Palade bodies of endothelial cells and rapidly mobilized and released during endothelial activation (46). Among lymphatic endothelial cells, it plays a more intriguing role in lymphangiogenesis (47). A previous study of Ang-2-deficient mice suggested that Ang-2 plays an important role in the development of lymph and angiogenic remodeling *in vivo* (48). A large dose of Ang-2 can promote lymphatic endothelial cell proliferation and cell survival (49). Based on histological findings, we speculate that the reason for the elevated level of Ang-2 is that similar to KHE, the normality and integrity of the vasculature in KLA patients are disrupted (50–52). The diseased spleen destroys many platelets and releases many factors, causing Ang-2 to accumulate at the lesion sites and be released into the serum. An elevated Ang-2 may promote the leakage of abnormal lymphatic vessels, resulting in pleural and peritoneal effusion. Nonetheless, further studies are needed to verify our hypothesis.

After splenectomy, splenic lesions were removed, followed by a decrease in platelet destruction and abnormal activation. Subsequently, there was a significant decrease in Ang-2 release. The previous studies have shown that a further decrease in Ang-2 during treatment is associated with a normalization of the platelet count and reduction of D-dimer (53). This achieved the goal of relieving conditions such as elevated platelet counts and decreased pleural effusion. Although the three patients had a good prognosis and we obtained some useful information, there are still some limitations in our research. These limitations include the unknown specific mechanism of splenectomy in the treatment of KLA, the unclear role of Ang-2 and platelets in vascular diseases, and our short-term follow-up. We will continue to explore the mechanisms and follow up with patients for more information.

In conclusion, due to the absence of comprehensive clinical guidelines and the complex nature of patient conditions, the treatment of KLA necessitates interdisciplinary collaboration. The interval between the onset and diagnosis of the disease is too long to lead to a good prognosis. In addition, KLA can impose a significant social burden on the patients and their families. Given the complexity of the disease and the long-term nature of treatment, good doctor‒patient communication and appropriate patient education are extremely essential. This can help them better comply with treatment plans and achieve greater benefits. Despite some progress, the treatment is still challenging. Therefore, there is an urgent need to determine the pathogenesis of KLA to explore and find new and more-effective drugs to help the patients. Despite the current difficulties in treatment, with the progress of genetic testing technology and the deepening of research related to lymphatic development, there will be more means to treat KLA in the future.

## Data Availability

The original contributions presented in the study are included in the article/Supplementary Material, further inquiries can be directed to the corresponding author.
